# The Importance of Edible Medicinal Mushrooms and Their Potential Use as Therapeutic Agents Against Insulin Resistance

**DOI:** 10.3390/ijms26020827

**Published:** 2025-01-19

**Authors:** Zsuzsanna Németh, Mariann Paulinné Bukovics, Liza Dalma Sümegi, Gábor Sturm, István Takács, Laura Simon-Szabó

**Affiliations:** 1 Department of Internal Medicine and Oncology, Semmelweis University, Koranyi S. u 2/a, 1083 Budapest, Hungary; sumegi.liza.dalma@semmelweis.hu (L.D.S.); takacs.istvan@semmelweis.hu (I.T.); 2 Independent Researcher, 1083 Budapest, Hungary; bukovics.mariann0710@gmail.com; 3 Directorate of Information Technology Basic Infrastructure and Advanced Applications, Semmelweis University, Üllői Út 78/b, 1082 Budapest, Hungary; sturm.gabor@semmelweis.hu; 4 Department of Molecular Biology, Semmelweis University, Tűzoltó u. 37–47, 1094 Budapest, Hungary; szabo.laura@semmelweis.hu

**Keywords:** medical mushroom, complementary therapy, IR, T2DM, signaling pathway

## Abstract

In addition to conventional treatments, there is growing interest in preventive and complementary therapies. Proper nutrition can prevent the manifestation of several chronic diseases such as obesity, diabetes, cardiovascular disease, and cancer, and can attenuate the severity of these diseases. Edible mushrooms have been used as nutrition and medicine for thousands of years. The spectrum and quantity of their medicinal compounds made them a widely investigated target both in basic research and clinical trials. The most abundant and medically important components are polysaccharides, terpenoids, phenols, and heterocyclic amines, but bioactive proteins, vitamins, including vitamin D, polyunsaturated fatty acids, and essential minerals are also important ingredients with noteworthy health benefits. Mushroom extracts have anti-diabetic, anti-hyperlipidemic, anti-inflammatory, antioxidant, cardioprotective, anti-osteoporotic, and anti-tumor effects and are well tolerated, even by cancer patients. In our previous review we detailed the molecular aspects of the development of type 2 diabetes, discussing the role of physical activity and diet, but we did not detail the role of medicinal mushrooms as part of nutrition. In this review, we aimed to summarize the most important medical mushrooms, along with their natural habitats, growing conditions, and components, that are presumably sufficient for the prevention and treatment of insulin resistance.

## 1. Introduction

In the last decade, there has been considerable interest in the efficiency of non-pharmacological treatments in disease prevention and their use as complementary treatments [1,2,3]. Sufficient physical activity and proper nutrition with adequate amounts of dietary components or, if needed, supplementation, are well-known regulators of homeostasis, and metabolic and immune processes, which enable the prevention and reduction of diseases and their progression [4,5,6,7,8,9,10].

Edible mushrooms are part of diets all over the world, and have also been used as medicine for thousands of years, mainly in Asia and Japan [11]. Their known pharmaceutical properties (i.e., anti-cancer, anti-inflammatory, antioxidant, and anti-diabetic) have made them a widely investigated target both in basic research and clinical trials [12,13,14]. The spectrum and quantity of medicinal compounds found in different edible mushrooms overlap to a large extent. The most abundant and medically important components are polysaccharides (among these α- and β-glucans), terpenoids, phenols, and heterocyclic amines [11,15]. Additionally, other important nutrients such as amino acids and proteins [16], unsaturated and polyunsaturated fatty acid (i.e., oleic and linoleic acids) [17], micronutrients like vitamin D (as ergosterol-the precursor of vitamin D2, or vitamin D2 itself) [12,18], vitamin B [19], and essential minerals (Zn, Fe, Mn, Ca, Mg), [12,20] as well as bioactive proteins (i.e., lectins, fungal immunomodulatory proteins (FIP), ribosome inactivating proteins (RIP), ribonucleases, laccases, and lentin) [21] are also important ingredients of edible mushrooms with noteworthy health benefits.

There is extensive knowledge in scientific literature from clinical, pre-clinical, and in vitro studies about the beneficial effects of mushrooms and their compounds against a variety of disorders and diseases. Reishi (*Ganoderma lucidum*) has several pharmacologically active compounds with antiviral, anti-inflammatory, antioxidant, cardioprotective, anti-osteoporotic, anti-tumor, neuroprotective, antidepressant, radio-protective effects and so on [12]. Mushroom extracts were well tolerated by cancer patients and found to improve quality of life (QoL) and immune outcomes (the molecular details of the effect on the immune system are not yet fully understood) and decrease anti-cancer treatment-related toxicities, thus increasing treatment adherence and improving outcomes without major adverse effects [13]. *Sparassis crispa*, which is sometimes called cauliflower fungus, has not only anti-cancer, but anti-inflammatory, anti-fungal, and antioxidant activities, based on the results of a meta-analysis where 33 randomized control trials (RCTs) were included [14]. Lentin from Shiitake (*Lentinula edodes*) inhibits HIV-1 reverse transcriptase and the proliferation of leukemia cells [22].

In our previous review, we detailed the molecular aspects of the development of type 2 diabetes (T2DM) along with possible preventive and complementary treatments, including physical activity and diet, but we did not discuss the role of medicinal mushrooms as part of nutrition [3]. In this review, we aimed to summarize those edible mushrooms that are considered and used as medical mushrooms due to their increased amount of medically effective composition. We detailed their components that are presumably sufficient for the prevention and treatment of insulin resistance and T2DM. We summarized their positive physiological roles and impact on specific and related signaling pathways. Additionally, we added supplementary information about the natural habitats and growing conditions of edible medical mushrooms (Supplementary Materials, Table S1).

## 2. Composition of Medical Mushrooms

Mushrooms are widely recognized for their dual role as both food and medicine, attributed to their rich composition of primary and secondary metabolites with notable health-promoting properties [23,24]. Key metabolites found in mushrooms included indole and phenolic compounds, carbohydrates, fatty acids, proteins, free amino acids, sterols, carotenoids, enzymes, and vitamins; alongside these, mushrooms are an important source of essential amino acids [25,26,27]. The indole (i.e., L-tryptophan, 5-hydroxy-L-tryptophan, tryptamine, 5-methyltryptamine, and melatonin) and phenolic derivatives (i.e., flavonoids, 4-hydroxybenzoic, ferulic, p-coumaric, protocatechuic, trans-cinnamic, and vanillic acid) of edible mushrooms have antioxidant properties [28].

The protein content of mushrooms is 12–35% of their dry weight and mushrooms are a highly digestible source of proteins, with a score of amino acids similar to milk and meat [29,30]. For instance, per 100 g of dry matter, mushrooms contain essential amino acids such as glutamic acid (130–240 mg), aspartic acid (91–120 mg), threonine (41–95 mg), arginine (37–140 mg), and valine (36–89 mg) [31]. However, some edible mushrooms are deficient in leucine, isoleucine, lysine, and tryptophan [32].

The fat content of mushrooms, measured on a dry matter basis, ranges from 1.1% to 8.3%, with an average of 4.0% [33]. This crude fat content comprises free fatty acids, mono-, di-, and triglycerides, sterols and their esters, and phospholipids [31,34].

Carbohydrates are another significant component, with their total content varying between 35% and 70% based on dry matter [30,33]. Digestible carbohydrates include glucose (<1%) and glycogen (5–10%). Additionally, mushrooms are a rich source of indigestible carbohydrates, such as oligosaccharides like trehalose and non-starch polysaccharides (NSPs) including chitin, β-glucans, and mannans [25,33].

Mushrooms are also valued for their vitamin content, including thiamine (vitamin B1), riboflavin (vitamin B2), niacin (vitamin B3), pantothenic acid (vitamin B5), biotin (vitamin B7), folate (vitamin B9), cobalamin (vitamin B12), ascorbic acid (vitamin C), and Vitamin D. Concentrations of certain vitamins per 100 g of dry matter include riboflavin (1.8–5.1 mg), folate (0.3–0.64 mg), and niacin (31–65 mg) [35,36].

The mineral composition of mushrooms is equally remarkable, with the ash content ranging from 7% to 17% based on dry matter [37]. Notable mineral concentrations per 100 g of dry matter include potassium (2700–4700 mg), phosphorus (500–1400 mg), magnesium (20–200 mg), zinc (4.7–9.2 mg), and copper (0.5–3.5 mg) [38,39].

Extensive research has been conducted on their therapeutic applications, revealing the significance of various bioactive compounds in their nutritional and medicinal benefits (Table 1) [21].

## 3. Medically Active Components with Positive Effects on Insulin Resistance

Insulin resistance (IR) is a clinical state [3], which is frequently diagnosed in obesity and T2DM [116,117]. It is developed under conditions when the human body continuously receives higher nutrient intake than it needs over a long period of time. In these circumstances the cells are unable to take up more glucose (impaired glucose tolerance or prediabetes), either because of a failure of insulin secretion or decreased insulin sensitivity [118]. The latter is partly the result of a negative feedback mechanism, where the nutrient overload, followed by the prolonged activation of insulin/Rheb/mTORC1/S6K1 signaling, induces Ser/Thr phosphorylation of IRS-1, resulting in its degradation blocking the activity of this pathway [119,120]. Although the terms “impaired glucose tolerance”, “glucose intolerance”, and “insulin resistance” suggest that only glucose homeostasis is involved in this physiological state, in fact, many other processes are involved.

**Figure 1:**
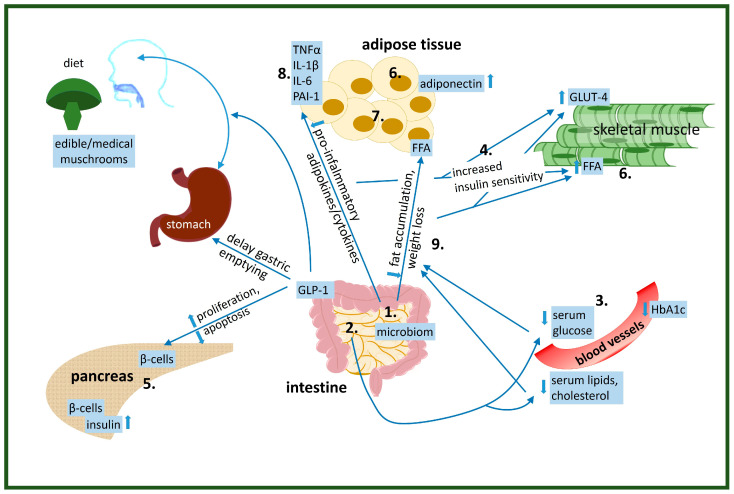
**Physiological effects of edible/medical mushroom components on the improvement of insulin resistance**: 1. altered gut microbiome, 2. decreased glucose absorption, 3. lowered serum glucose levels, 4. increased glucose uptake by the cells, 5. increased/decreased insulin production by β-cells in pancreas, 6. altered lipid metabolism-increase utilization of FFA as an energy source in muscle, 7. altered adipose tissue function, 8. reduced pre-inflammatory cytokine levels, 9. reduced/induced weight loss.

Several in vivo and human studies discuss the beneficial effects of various doses of compounds from edible mushrooms on insulin resistance [121]. Important parts of glucose and lipid homeostasis where the compounds of medical mushrooms can effectively alter IR according to our recent scientific knowledge are listed below (Figure 1):

1. alter gut microbiom
2. decrease glucose absorption
3. lower serum glucose levels
4. increase glucose uptake by the cells
5. increase/decrease insulin production by β-cells in pancreas
6. alter lipid metabolism-increase utilization of FFA as energy source in muscle
7. alter adipose tissue function
8. reduce pre-inflammatory cytokine levels
9. reduce/induce weight loss

### 3.1. Altered Gut Microbiota

Mushrooms are considered a potential source of prebiotics due to their non-digestible, water-soluble, and insoluble polysaccharides, which stimulate the growth of beneficial bacteria in the colon [41]. The importance of gut microbiota in the development of obesity, T2DM, and insulin resistance has been known for a long time [122,123] but has recently come back into focus again as a potential target for improving insulin resistance using a comprehensive multi-omics strategy in humans [124]. Beneficial gut microbiota have important physiological functions, including the development of a proper immune system, regulation of intestinal barrier and endocrine function, and modulation of glucose and lipid homeostasis [125]. Moreover, it is not only the proportion of different bacterial genera that is important in the microbiota. The diversity of these bacteria is also important, as more of them can better digest food and produce essential amino acids or vitamins, thus reducing the risk of many diseases by increasing the absorption of nutrients necessary for maintaining health [126,127,128].

Microbiota derived metabolites such as short-chain fatty acids (SCFAs) acetate, propionate, and butyrate can suppress fat accumulation and improve energy expenditure in the liver and muscle through the G-protein-coupled receptor 43 (GPR43) [110]. However, SCFA supplementation itself significantly increased the GPR43 level in adipose tissues, with subsequent positive effects on altered gut microbiota, i.e., increased proportion of *Bacteroidetes*, decreased *Firmicutes*, and altered body composition, suggesting a positive feedback loop between SCFAs and gut microbiota [129]. Additionally, these SCFAs replacing carbohydrates may serve as an energy source and thus decrease insulin/IGF-1 signaling and further improve health [130].

The water extract of *Ganoderma lucidum* altered gut microbiota composition by increasing the variety of the above mentioned beneficial bacterial species (i.e., *Parabacteroides goldsteinii*, *Bacteroides* spp., *Anaerotruncus colihominis*, *Roseburia hominis*, *Clostridium methylpentosum*, *Eubacterium coprostanoligenes*), which correlated with improved body composition by reducing obesity [71]. Additionally, it restored the expression level of proteins, which are important for maintaining intestinal tight junction integrity. This alteration prevented the translocation of pro-inflammatory endotoxins into the circulation in high-fat diet-fed mice [48,71]. Fungal polysaccharides cannot be degraded in the human stomach and small intestine; these can be digested into SCFAs by intestinal bacteria in the colon, resulting in the induced secretion of GLP-1 from intestinal cells [131,132]. GLP-1 then influences the central nervous system, leading to reduction in appetite; it affects muscles, adipose tissue, liver, and stomach—in the latter it delays gastric emptying [48].

Similarly to the water extract of *Ganoderma lucidum*, *Morchella esculenta* polysaccharides also improved the composition of gut microbiota in a T2DM mice model, where the majority of the flora reverted to normal (i.e., increased abundance of *Lactobacillaceae*, *Lachnospiraceae*, and *Enterobacteriaceae* and decreased abundance of *Staphylococcaceae* and *Corynebacteriaceae*). Furthermore, similarly to the aqueous extract of *Ganoderma lucidum*, polysaccharides from *Morchella esculenta* restored intestinal permeability by inducing tight junction (ZO-1, occludin) protein levels [102].

An aqueous extract of *Antrodia cinnamomea* significantly increased the proportion of bacteria with anti-inflammatory properties in the normal intestinal flora (included the reduced *Firmicutes*/*Bacteroidetes* ratio), and also the levels of tight-junction protein ZO-1 and occludin, maintaining intestinal barrier integrity [56]. All of these resulted in reduced endotoxemia and chronic inflammation in treated mice fed a high-fat diet compared to untreated mice. Additionally, the antimicrobial peptide Reg3g and lysozyme C also were significantly elevated after the treatment in the ileum of high-fat diet-fed animals compared to their non-treated controls [56]. These antimicrobial effectors play an important role in maintaining homeostasis of gut microbiota [133], and their reduced expression was also demonstrated in mice fed a high-fat diet [134].

Extracts of *Pleurotus ostreatus* and *Pleurotus eryngii*, mainly containing glucans, were able to stimulate the probiotic growth of *Lactobacillus* ssp. and *Bifidobacterium* ssp., among others. [108]. Although the changes in intestinal microbiota were not evaluated in a double-blind, randomized, controlled crossover trial, oyster mushroom (*Pleurotus ostreatus*) powder rich in β-glucans, similar to the aforementioned polysaccharides from other mushrooms, increased the levels of GLP-1 and subsequently decreased appetite [112].

### 3.2. Decreased Glucose Absorption

The α-amylase and α-glycosidase enzymes are responsible for degrading carbohydrates into glucose, and through their activity they increase glucose absorption. The intracellular polysaccharides of *Hericium erinaceus* significantly inhibited the enzymes α-amylase and α-glucosidase, thus reducing glucose absorption [87]. Triterpenoids isolated from *Ganoderma lucidum* with chloroform or ethanol inhibited α-glucosidase enzymes and thus reduced glucose absorption from the intestine [76,77,78]. Similarly, the triterpenoids inotolactone A and B, extracted from submerged cultures of *Inonotus obliquus*, have inhibitory effects on α-glucosidase activity [96].

### 3.3. Lowered Serum Glucose Levels

The 500 and 750 mg/kg ethanol extract of White bottom mushroom *Agaricus bisporus* effectively reduced blood glucose levels in alloxan-induced diabetic rats compared to controls [40]. Consumption of 2 g/kg *Agaricus bisporus* per day was associated with significantly reduced glucose levels compared to controls in a human study [43]. Powder from this mushroom also significantly improved the glycemic index of T2DM patients after treatment in a randomized control trial [44]. Treatment with *Morchella esculenta* polysaccharides significantly decreased fasting glucose levels compared to the non-treated groups in T2DM mouse model [102,103]. Supplementation with an aqueous extract of *Antrodia cinnamomea* similarly reduced fasting glucose levels, as demonstrated by an oral glucose tolerance test, in treated mice fed a high-fat diet compared to their untreated counterparts [56]. In addition, administration of *Antrodia cinnamomea* powder at an optimal dose of 200 mg/kg body weight, which contains eburicoic acid, dehydroeburicoic acid, sulphurenic acid, dehydrosulphurenic acid, and ergostatrien-3β-ol, induced a significant decrease in plasma glucose levels at 30 and 60 min after administration compared to a control Wistar rat group [58]. A *Grifola frondosa* GF5000 water extract fraction (Mw > 5000D) significantly reduced fasting serum glucose levels in treated rats compared to diabetic controls [80]. Boletus polysaccharides significantly decreased fasting blood glucose levels in a treated group compared to controls in a T2DM rat model, in which the effect was comparable to metformin [65]. The FXM fraction of *Grifola frondosa* containing β-glucans (also found in cereals), which has been shown to be anti-diabetic according to several studies [135,136,137,138,139], significantly lowered the circulating glucose concentration in insulin-resistant KK mice at 8–12 and 16–18 h in an acute study, and on day 4 and 7 in a chronic study after oral gavage compared to controls [86]. *Flammulina velotipes* polysaccharides significantly decreased fasting serum glucose levels in treated compared to non-treated diabetic groups of mice [69]. After feeding streptozotocin-induced diabetic rats with *Pleurotus ostreatus* mushroom, their serum glucose levels decreased significantly [111,113,114], similarly to the clinical trial, where after consumption of *Pleurotus ostreatus* mushroom, a significant decrease in fasting and postprandial serum glucose level was observed both in healthy volunteers and diabetic patients [115]. Phthalaldehyde derivatives hericenal A, B, and C, from submerged cultures of *Hericium erinaceus*, have potential anti-hypergylcemic effects in diabetic patients [140]. Similarly, D-threitol, D-arabinitol, palmitic acid, and α-D-glucan from *Hericium erinaceus* also resulted in anti-hyperglycemic effects in diabetic rats [87,90]. Both methanol and aqueous extracts of *Hericium erinaceus* significantly reduced fasting serum glucose levels in streptozotocin-induced diabetic rats [90,91]. Moreover, extracellular polysaccharides from the Turkey tail mushroom after 4 weeks administration attenuated the elevation of blood glucose levels in a dose-dependent manner in T2DM rats [68].

### 3.4. Increased Glucose Uptake by Cells

SX-fraction (SXF) of *Grifola frondosa* significantly increased the glucose uptake in rat skeletal muscle L6 cells [81] and resulted in around a 30–63% decline in fasting blood glucose levels after treatment in diabetic patients [81,82]. Additionally, 8 weeks of treatment with *Grifola frondosa* inhibited the rise in blood glucose levels in spontaneously diabetic mice. This effect was further confirmed in a crossover experiment (an experiment where at the half-term of the treatments, groups were switched) [83]. *Grifola frondosa* polysaccharides induced significant glucose consumption in insulin-resistant HepG2 cells after 24 h of treatments compared to non-treated controls, suggesting improved insulin resistance [84]. A ReishiMax supplement containing polysaccharides and triterpenes from *Ganoderma lucidum* significantly increased glucose uptake by adipocytes through the activation of AMP-activated protein kinase (AMPK) [75]. Extracellular polysaccharopeptides obtained from *Trametes versicolor* induced glucose uptake in insulin-resistant HeG2 cells through activation of AMPK, insulin receptor substrate 2 (IRS-2), and increased levels of glucose transporter-1. Additionally, this compound significantly increased the glycogen content, suggesting that extracellular polysaccharopeptides regulate glucose uptake and glucose homeostasis in an insulin-independent manner [67].

### 3.5. Insulin Production and Effects on β-Cells in Pancreas

The alteration of gut microbiota by increased SCFAs subsequently induced the expression of GLP-1, which promoted proliferation and inhibited apoptosis in pancreatic β-cells [141,142].

Treatments with polysaccharide extracted from *Morchella esculenta* decreased fasting serum insulin levels in a T2DM mouse model [102]. Water extract from *Antrodia cinnamomea* similarly decreased fasting insulin levels in treated compared to untreated high-fat diet-fed mice, as confirmed by an insulin tolerance test [56]. Moreover, *Antrodia cinnamomea* powder containing eburicoic acid, dehydroeburicoic acid, sulphurenic acid, dehydrosulphurenic acid, and ergostatrien-3β-ol at an optimal dose of 200 mg/kg significantly increased plasma insulin levels at 30 min, and significantly decreased the HOMA-IR 60 min after administration compared to the control Wistar rat group [58]. Treatments with *Grifola frondosa* inhibited the rise in insulin levels in spontaneously diabetic mice compared to untreated groups, which was also confirmed by crossover experiments [83]. The FXM fraction containing β-glucan from *Grifola frondosa* significantly lowered the circulating insulin concentration in insulin-resistant KK mice on day 4 and 7 in a chronic study after oral gavage compared to controls [86]. *Flammulina velotipes* polysaccharide, similarly, significantly decreased fasting serum insulin levels after treatment compared the non-treated pairs [69]. The Mukitake mushroom *Panellus serotinus* alleviated the sever hyperinsulinemia in db/db mice after a 4 week feeding period, which was observed in control-fed db/db mice [105].

### 3.6. Altered Lipid Metabolism-Increase Utilization of FFA as an Energy Source in Muscle

Supplementation with the high molecular weight polysaccharide fraction of *Pleurotus eryngii* significantly increased the gene expression levels of LDL receptor (*LDLR*) and GPR43 in the liver and adipose tissue of mice fed a high-fat diet, respectively [143]. This finding supports the idea that microbiota-derived SCFA metabolites, through activation of *GPR43* in adipocytes, prevent fat accumulation and increased energy utilization in other tissues, including muscle, where they improve glucose tolerance [110,144]. SCFAs can serve as an energy source instead of carbohydrates, thereby reducing insulin/IGF-1 signaling and improving health [106]. *Morchella esculenta* polysaccharides showed cholesterol-lowering effects by inducing the expression of *CYP7A1* and *LDLR* and down-regulating HMG-CoA [103,104]. Fibers from food such as chitosan from mushrooms are not digested in the stomach nor in the small intestine. Thus, besides their bulking effects (i.e., delayed gastric empty and inducing satiety), binding dietary fats and bile acids inhibiting their absorption/enterohepatic circulation, consequently, reduce blood lipid and cholesterol levels [145,146]. *Ganoderma lucidum* polysaccharides reduced lipogenic gene expression in a dose-dependent way in high-fat diet-fed mice compared to non-treated pairs [71]. Water extract of *Antrodia cinnamomea* significantly decreased serum triglyceride levels in treated compared to non-treated high-fat diet-fed mice [56], and also significantly decreased triglyceride, LDL-C and total cholesterol in treated compared to non-treated obese mice [147]. Similarly, powder of *Antrodia cinnamomea* at an optimal dose of 200 mg/kg significantly decreased plasma FFA levels 60 min after administration compared to control Wistar rats [58]. In a human study, consumption of 2 g/kg *Agaricus bisporus* per day was associated with significantly reduced levels of total cholesterol, LDL-C, and TG and increased levels of HDL-C compared to controls [43]. Treatments with *Grifola frondosa* inhibited the rise in triglyceride levels compared to non-treated groups of spontaneously diabetic mice, which was also confirmed in crossover experiments [83]. Similarly, the GF5000 fraction of this mushroom significantly decreased total serum cholesterol and LDL-C levels in treated diabetic rats compared to diabetic controls [80]. *Boletus* polysaccharides significantly decreased total cholesterol, triglyceride, and LDL-C-cholesterol levels in treated groups compared to control T2DM rats, while HDL-C-cholesterol significantly and inversely changed after treatment. In addition, all levels were similar to normal untreated controls, indicating that boletus polysaccharides can be an effective alternative in the reduction in serum lipids [65]. *Flammulina velotipes* polysaccharides significantly decreased total cholesterol, triglyceride, LDL-C, and FFA and increased HDL-C levels in treated groups compared to non-treated streptozotocin-induced mice [69]. Extracts from *Hericium erinaceus* have potential anti-hypecholesterolemic effects in diabetic rats [90,92,93]. D-threitol, D-arabinitol, palmitic acid, and α-D-glucan from Hericium erinaceus, and both the methanol and aqueous extracts, significantly reduced the elevation of serum TG, total cholesterol [69], and additionally LDL-C, and increased HDL-C levels in treated compared to non-treated diabetic rats [91]. Extracts from *Pleurotus ostreatus* mushroom significantly decreased total cholesterol, TG, and LDL-C levels in streptozotocin-induced diabetic rats, while HDL-C levels were significantly increased after treatments compared to diabetic controls [111]. Similarly, a 25-week treatment with *Lentinula edodes* mushroom significantly decreased serum TG, total cholesterol, and LDL-C with an increased ratio of HDL-C/LDL-C in high-fat diet-fed C57BL/6 mice compared to controls [97]. Extracellular polysaccharides from Turkey tail mushroom after 4 weeks of administration attenuated the elevation of serum TG levels in a dose-dependent manner in T2DM rats [68].

### 3.7. Altered Adipose Tissue Function

It is well known that genes responsible for fatty acid synthesis (i.e., acetyl-CoA carboxylase-1 (ACC-1), fatty-acid synthase (FAS), sterol regulatory element-binding protein-1c (SREBP-1c)) are upregulated, while those regulating catabolism (i.e., PPARγ co-activator 1α (PGC-1α)) are downregulated in high-fat diet-fad mice [148]. Overexpression of *GPR43* in the adipose tissue of high-fat diet-fed mice resulted in reduced white adipose tissue and body weight, and also in improved glucose tolerance [110,144].

Water extract of *Antrodia cinnamomea* attenuated ACC-1, FAS, and SREBP-1c and enhanced PGC-1α gene expression in treated compared to non-treated high-fat diet-fed mice [56]. A ReishiMax supplement containing polysaccharides and triterpenes from *Ganoderma lucidum* inhibited adipocyte differentiation by suppressing peroxisome proliferator-activated receptor-γ (PPAR-γ), SREBP-1c, and CCAAT/enhancer binding protein-α (C/EBP-α) transcription factors. Moreover, it suppressed FAS, acyl-CoA synthetase-1 (ACS1), fatty acid binding protein-4 (FABP4), fatty acid transport protein-1 (FATP1), and perilipin enzymes, which are responsible for lipid synthesis, transport, and storage [75]. Water extract of *Ganoderma lucidum* significantly reduced subcutaneous as well as liver fat accumulation by the alteration of gut microbiota in high-fat diet-fed mice. Additionally, it decreased the infiltration of anti-F4/80 and CD11b/CD11c-positive macrophages into the liver/adipose tissues and also increased the levels of Treg cells in the liver and adipose tissue in treated groups compared to non-treated mice [71]. Moreover, improvement in gut microbiota composition also resulted in a significantly decreased production of pro-inflammatory cytokines such as TNF-α, interleukin-1 beta (IL-1β), interleukin-6 (IL-6), and plasminogen activator-inhibitor 1 (PAI-1) in adipocytes [48,71]. *Antrodia cinnamomea* similarly altered adipocyte function, as seen in the significantly decreased pro-inflammatory marker TNF-α, IL-1β, IL-6, and leptin levels, while adiponectin levels significantly increased in the treated groups compared to the non-treated high-fat diet-fed mice [56]. Twelve weeks of treatment with extract from *Agaricus blazei Murill* resulted in significantly decreased insulin levels and HOMA-IR in diabetic patients in parallel with significantly increased adiponectin levels [50]. The Mukitake mushroom *Panellus serotinus* significantly increased the serum adiponectin levels of db/db mice after a 4 week period of treatment compared to control-fed db/db mice. In addition, it also significantly decreased the serum levels of monocyte-attracting protein 1 (MCP1) [105].

### 3.8. Reduced Pro-Inflammatory Cytokine Levels and Immunomodulatory Properties

A decreased production of pro-inflammatory cytokines such as TNF-α, IL-1β, IL-6, and PAI-1 was detected after treatments of water extract of *Ganoderma lucidum* compared to non-treated high-fat diet-fed mice [48,71]. Similarly, a 5 g/day extract of α-glucans from *Agaricus bisporus* significantly decreased TNF-α levels compared to placebo controls [42]. *Morchella esculenta* polysaccharides also significantly reduced serum pro-inflammatory cytokine levels in a T2DM mouse model [102]. Furthermore, water extract of *Antrodia cinnamomea* decreased the serum levels of TNF-α, IL-1β, and IL-6 in treated mice compared to non-treated high-fat diet-fed mice [56]. In addition, *Boletus* polysaccharide treatment significantly decreased nuclear factor kappa B (NF-κB) expression, which is responsible for pro-inflammatory cytokine production, compared to the control T2DM group; the expression of TNF-α also significantly decreased to normal levels [65]. *Flammulina velutipes* polysaccharides have immunomodulatory and anti-inflammatory properties, based on recent studies [70]. Secondary metabolites from *Hericium erinaceus*, which have poor water solubility, have immunomodulatory properties as well [88,89]. A 25-week treatment with *Lentinula edodes* mushroom was associated with immunomodulation observed in an increased CD4^+^/CD8^+^ lymphocyte ratio and in a shift from pro- to anti-inflammatory cytokine production compared to high-fat diet-fed C57BL/6 control mice [97].

### 3.9. Induced/Reduced Weight Loss

A study investigating β-glucan supplementation in high-fat diet-fed induced obese rats for 6 weeks found that β-glucan, which is predominantly found in mushrooms and cereals, has beneficial therapeutic potential against obesity [149]. The treatment significantly reduced body weight and attenuated obesogenic markers such as hyperglycemia, dyslipidemia, and insulin resistance by altering the expression of PPAR-γ, SREBP-1c, FAS, HMG-CoA reductase, and Fab-4 in high-fat diet-fed induced obese rats. Water extract of *Ganoderma lucidum* induced significant weight loss in obese animals by altering gut microbiota composition, with this effect being primarily attributed to high molecular weight polysaccharides (>300 kDa) [48,71]. Concordantly, water extract of *Antrodia cinnamomea* induced a significant loss of body weight of around 10% in treated compared to untreated high-fat diet-fed mice [56]. SCFA supplementation altered gut microbiota, with an increased proportion of *Bacteroidetes* and decreased proportion of *Firmicutes*, and significantly increased GPR43 levels in adipose tissue, preventing high-fat-diet-induced obesity in mice [129]. Treatments with *Grifola frondosa* inhibited weight gain in spontaneously diabetic mice compared to non-treated groups, and this was confirmed by crossover experiments [83]. In a T2DM rat model, body weight was significantly decreased compared to the normal group but *Boletus* polysaccharide supplementation was able to revert this, resulting in attenuated weight loss in the treated group [65].

## 4. Supposed Signaling Mechanisms Targeted by Medicinal Mushroom Components

Increased endotoxin in the serum as the result of a leaky gut [150,151] induced JNK and NF-κB signaling activation [56], which inactivated IRS-1 by its inhibitory phosphorylation, leading to decreased insulin signaling and insulin resistance [152,153]. *Ganoderma lucidum* and *Antrodia cinnamomea* extracts could reduce endotoxemia, which could revert insulin signaling through IRS-1 reactivation [56,71]. Additionally, administration of *Antrodia cinnamomea* powder containing eburicoic acid, dehydroeburicoic acid, sulphurenic acid, dehydrosulphurenic acid, and ergostatrien-3β-ol, at an optimal dose of 200 mg/kg induced significant induction of insulin signaling with increased PI3K, IRS-1, and GLUT-4 levels compared to control Wistar rats [58].

Water-extracted mushroom polysaccharides increased SCFA levels in the intestine, inducing GLP-1 production, which led to increased expression of IGF-2/IGF-1R in an autocrine manner. This activation of the ligand-bound receptors then activated the Akt pathways in pancreatic β-cells, and, in a parallel process, inhibited apoptosis and induced proliferation in these β-cells [141].

SXF of *Grifola frondosa* significantly increased glucose uptake due to the reactivation of insulin signaling. In detail, SXF treatments decreased inhibitory serine phosphorylation of IRS-1 while increasing activating tyrosine phosphorylation, resulting in Akt activation and putative GLUT4 translocation to the plasma membrane [81]. *Pleurotus ostreatus* mushroom decreased hyperglycemia in streptozotocin-induced diabetic rats through increased p-AMPK levels and expression of GLUT4 in muscles and adipose tissues [113]. In insulin-resistant HepG2 cells, polysaccharides obtained from *Grifola frondosa* significantly increased Akt phosphorylation and thus inhibited glycogen synthase kinase-3 (GSK-3), the inhibitor of glycogen synthase, and subsequently increased glucose uptake and glycogen synthesis [84]. Moreover, novel heteropolysaccharides (GFP-N) from *Grifola frondosa* caused hypoglycemic effects in insulin-resistant HepG2 cells via activation of IRS-1, PI3K, and GLUT4 signaling and inhibition of JNK/p38 pathways [85]. Water extract of *Coriolus versicolor* significantly increased the mRNA expression of PI3K, p-Akt, Akt, p-p38 mitogen-activated protein kinase (MAPK), and p38 MAPK in rat skeletal muscles in vivo, in which signaling was reported to upregulate GLUT4, and thus reduce insulin resistance [66].

The molecular background of the anti-hyperlipidemic effects of *Flammulina velutipes* was demonstrated by the activation of the PI3K/Akt pathway in liver, where p-PI3K, p-Akt, GLUT4, and IRS-1 levels were significantly up-regulated after high-dose polysaccharide intervention in diabetic mice [69].

The GF5000 fraction from *Grifola frondosa* decreased pro-inflammatory cytokine levels, likely by suppressing the TLR4/MyD88/NF-κB pathway [80].

The signaling mechanisms targeted by the medicinal mushroom components described above are listed in Table 2.

## 5. The Role of the Vitamin D_2_ Component in Medical Mushrooms in the Context of Insulin Resistance

Vitamin D has a fundamental role in calcium homeostasis and bone metabolism [9]. However, its pleiotropic effects on immunity, cell growth, differentiation, and energy metabolism are now widely known due to the results of extensive studies [154,155] following the discovery of 25(OH)D [156], and then its hormonally active form, 1,25(OH)_2_D calcitriol [157].

Vitamin D is one of the fat-soluble vitamins and has two forms, ergocalciferol (vitamin D_2_) and cholecalciferol (vitamin D_3_) [158,159]. Plants and mushrooms form vitamin D_2_ from its ergosterol precursor by ultraviolet B irradiation (UVB) [18], while vitamin D_3_ is synthesized in the epidermis from 7-dehydrocholesterol, also by UVB. Around 80% of vitamin D is produced by our skin and the remaining 20% is provided by our nutrition [160]. The content of ergosterol and vitamin D_2_ varies among different mushroom species [161].

UVB-exposed *Agaricus bisporus* consumption significantly increased serum 25(OH)D levels in patients to the same extent as supplementation with ergocalciferol or cholecalciferol [42]. Ergosterol did not alter the 25-hydroxylation process in either the HepG2 cells or the liver of ergosterol-supplemented mice, and the levels of 25(OH)D in serum and tissues were unchanged compared with cholecalciferol supplemented groups. It also did not change the concentration of 1,25(OH)_2_D and 24,25(OH)_2_D in the serum of treated animals compared to cholecalciferol supplementation. In addition, ergosterol did not alter liver lipid concentrations compared with cholecalciferol supplementation in treated mice [162]. In our previous review, we briefly discussed the positive role of vitamin D in insulin resistance [3], we discuss this in more detail below.

Primarily, the active metabolite, the hormone calcitriol (1,25(OH)_2_D), mediates the biological effects of vitamin D in organisms by binding to the vitamin D receptor (VDR) [2,154,158,163,164,165]. Upon binding to calcitriol, the nuclear receptor VDR forms a heterodimer with the retinoic acid X receptor (RXR) to enhance or inhibit the transcription of thousands of genes [154,163]. Although a membrane-associated VDR is also known to initiate membrane-signaling cascades [166], and non-genomic effects of 1,25(OH)_2_D do not necessarily require VDR [167], the physiology of the genomic and non-genomic effects overlaps to a large extent [154]. Interestingly, not only calcitriol, but also the inactive 25(OH)D form can bind to VDR, ensuring the intracrine effects of vitamin D in addition to 1,25(OH)_2_D produced locally and specifically in various tissues [168,169,170]. The presumed role of vitamin D was primarily probably the regulation of energy metabolism, and later it acquired new functions, namely the modulation of the innate and adaptive immunity, and the regulation of calcium and bone homeostasis [164].

Metabolic syndrome (MetS) is a complex metabolic disorder characterized by four main factors: hypertension, dyslipidemia, abdominal obesity, and IR, among others [3,118,171]. Several studies have shown that vitamin D levels (25(OH)D) are inversely associated with MetS factors [2,172,173]. Vitamin D deficiency has been linked to the earlier-onset and higher severity of T2DM, due to abnormal secretion of insulin and immune dysfunction [155]. However, vitamin D may prevent pancreatic β-cell destruction and the incidence of autoimmune diabetes, likely through the inhibition of pro-inflammatory cytokine release [174]. Furthermore, vitamin D supplementation was effective in improving T2DM-related conditions such as hyperglycemia and increased hemoglobin A1c (HbA1c) levels [175,176].

Vitamin D supplementation also significantly elevated the levels of SIRT1 and SIRT6, which play important roles in glucose homeostasis by increasing insulin secretion, inhibiting gluconeogenesis and lipogenesis and suppressing obesity-induced inflammation and insulin resistance [177,178,179]. Vitamin D increased glucose uptake by inducing the insulin-independent SIRT1/AMPK/IRS1/GLUT4 signaling pathway [180]. Additionally, vitamin D increased insulin sensitivity, presumably through an increase in Ca2^+^ influx, which stimulated insulin receptor expression, activation of the GLUT-4 glucose transporter, and activation of peroxisome proliferator-activated receptor delta (PPAR-δ) [181,182].

In addition to glucose homeostasis, vitamin D also supports optimal lipid homeostasis through increased expression of adiponectin and activation of AMPK in adipocytes [2,183]. Adiponectin has positive effects on both glucose and lipid metabolism, increasing glucose and FFA utilization in skeletal muscle and reducing blood glucose levels, as well as increasing HDL-C while decreasing TG levels [184]. Adiponectin is similarly important in balancing immune processes due to its anti-inflammatory properties [185,186].

Vitamin D also plays an important role in the direct regulation of innate and adaptive immune systems, through VDR, which is expressed in almost all immune cells [187]. Vitamin D modulates immune reactions by inducing anti-inflammatory cytokine production [70,188,189,190], through the suppression of TLR2 and TLR4 proteins and NF-κB signaling [187]. In addition, but partly through these aforementioned processes, it decreases low-grade chronic inflammation coexisting with IR [160]. Furthermore, vitamin D, as an epigenetic regulator, maintains the expression of DNA demethylases and thus prevents hypermethylation, which is an important characteristic of T2DM patients [191,192].

It is widely known that hyperglycemia causes oxidative stress through the overproduction of reactive oxidative species (ROS); however, vitamin D can protect cells from ROS overproduction and control mitochondrial respiration [193,194,195]. Additionally, in high-glucose-treated adipocytes, vitamin D inhibited oxidative stress as well through SIRT1/AMPK/GLUT4 signaling [196].

Calcification or vitamin D intoxication is an important consideration when using vitamin D supplementation as a preventive or complementary therapy. It should be noted that excessive exposure to sunlight cannot cause intoxication, as both the inactive and active form produced by the skin are photolabile and thus easily converted to biologically inactive products [197,198]. It is also important to note that levels of the inactive 25(OH)D form did not correlate with calcification related measures [199,200]. However, it is assumed that vitamin D_2_ is less effective in bone metabolism because it is not bone selective [201]. Moreover, patients with chronic kidney disease [202], vitamin D oversupply, and patients with vitamin D deficiency [203] with certain related health issues have increased risk, which may lead to calcification. Therefore, in these cases, additional laboratory parameters, i.e., measurement of serum calcium, phosphate, parathyroid hormone, creatinine, and alkaline phosphatase levels, should also be considered [10]. However, it is also important to know that convincing molecular evidence suggests that calcification is not only an active process but may also be reversible if treated at an early stage [202].

## 6. Summary

Edible mushrooms, including medical mushrooms, are an important part of nutrition. They are good source of fiber, vitamins, amino acids, and trace elements, but the most investigated are the water-soluble composites, polysaccharides. Polysaccharides have complex effects on the human body. They favorably modulate the intestinal microbiota, glucose, and lipid homeostasis, as well as the immune system, the combined effect of which can reduce insulin resistance. Thus, these effects make mushrooms, especially medicinal mushrooms, a potential part of complementary therapy for obesity and related diseases, such as type 2 diabetes.

## Figures and Tables

**Table 1:** **Medically active components of medical mushrooms**. CAT—catalase; FFA—free fatty acid; GLUT4—glucose transporter type 4; GLP-1—glucagon-like peptide-1; GPR43—G-protein-coupled receptor 43; GSH—glutathione; GSH-Px—glutathione peroxidase; HDL-C—high-density lipoprotein cholesterol; HMG-CoA—3-hydroxy-3-methylglutaryl-CoA; HOMA-IR—Homeostatic Model Assessment for Insulin Resistance; IKKβ—inhibitor of κB kinase β; IκB—inhibitor of κB; IgA—immunoglobulin A; LDL-C—low-density lipoprotein cholesterol; MCP1—monocyte chemoattractant protein 1; MDA—malonaldehyde; NF-κB—nuclear factor kappa B; PI3K—phosphoinositide 3-kinase; PSA—prostate specific antigen; SCFAs—short-chain fatty acids; SHR—spontaneously hypertensive rats; SOD—superoxide-dismutase; TG—triglyceride; TGF-β1—transforming growth factor beta 1; TNF-α—tumor necrosis factor α; ZFR—Zucker fatty rats.

*Latin Name*/Common Name	Active Ingredients	Physiological Effects Cellular/Molecular Effects	References
*Agaricus bisporus*/White bottom mushroom	flavonoids, alkaloids, terpenoids, and saponins	decrease blood glucose and MDA levels as well as increase SOD activity	[11,40]
	polysaccharides (i.e., Xylose, Fructose, Mannose, Glucose, Sucrose, Trehalose)	induce the growth of beneficial gut bacteria	[41]
	flavonoids, ergosterol and vitamin D_2_, ergothionine, glucans, chitin, myricetin quercetin, kaempferol, catechin, naringenin, resveratrol, anthocyanins, gallate, and tannic acid	hypoglycemic, decrease LDL-C, TG and body weight, increase HDL-C increase adiponectin, increase serum IgA, decrease TNF-α, anti-cancer effect (decreased PSA, reduced odds of ovarian cancer)	[42,43]
	hot air-dried powder	hypoglycemic, decrease LDL-C level	[44]
	fruiting body	anti-obesity	[30,45]
	ergothioneine	antioxidant, cytoprotective	[46,47]
*Agaricus brasiliensis* (*Agaricus blazei Murrill*)	statin (Lovastatin)	inhibit lipid synthesis, through inhibition of HMG-CoA reductase, hypoglycemic and anti-anemic action	[48,49]
	gliclazide	lowers HOMA-IR index increase plasma adiponectin	[50]
		anti-cancer effects	[51,52,53]
*Amillariella mellea*	polysaccharides	lower fasting blood glucose, improve glucose intolerance and insulin resistance-decrease serum triglycerides, inhibit lipid accumulation	[54]
*Antrodia cinnamomea* (Syn. *Antrodia camphorata*)	polysaccharides, triterpenoids, steroids, phenolic components, cordycepin, sesquiterpene, adenosine, ergosterol, and maleic/succinic acid derivatives	weight loss, reduce fat accumulation, reduce serum triglycerides, reduce pro-inflammatory markers, decrease leptin and increase adiponectin levels, maintain gut barrier through increased expression of tight junction proteins, prevent diabetes-induced male reproductive dysfunction	[55,56,57]
	eburicoic acid, dehydroeburicoic acid, sulphurenic acid, dehydrosulphurenic acid, and ergostatrien-3β-ol	hypoglycemic effect increase insulin level decrease HOMA-IR decrease FFA increase insulin signaling	[58]
*Auricularia auricular*	polysaccharides polyphenols	maintain glucose level, attenuate inflammatory signaling	[59,60]
		increase glycogen, C-peptid, GLP-1 levels	[61]
		reduce total cholesterol and LDL-C-cholesterol	[62]
		anti-cancer activity	[63]
	flavonoids and phenolic acids	inhibition of α-amylase activity	[64]
*Boletaceae Boletales*/ Boletes	tocopherol, quinic acid, hydroxyl benzoic acid, polysaccharide	antioxidant (CAT, SOD, GSH) anti-inflammatory (decreases NF-κB, TNF-α level) hypoglycemic	[11,65]
*Coriolus versicolor* or *Trametes versicolor*/Turkey Tail	water extract	decreases blood glucose level, increases glucose consumption, increases GLUT4 expression and translocation	[66]
	extracellular polysaccharopeptides	elevates cellular glucose uptake to regulate glucose homeostasis in an insulin-independent manner	[67]
		anti-hyperglycemic anti-hypertriglyceride alleviate oxidative stress (decrease lipid-peroxidation, increased SOD, GSH)	[68]
*Flammulina velutipes*/Enoki mushroom	polysaccharide	immunomodulation, anti-inflammatory effect anti-hypertension	[69,70]
	flammulinolide	anti-tumor	
	enokipodin proflamin	reduces blood glucose and insulin level	
	other polysaccharides	decreases total cholesterol, triglyceride, LDL-C-C and FFA and increase HDL-C-C level by activation of PI3K/Akt signaling pathway in the liver	
*Ganoderma lucidium*/ lingzhi or reishi	polysaccharides,	improve gut micribiota composition	[49,71,72]
	β-glucans, lectines, eritadenin, triterpenes, sterols,	reduce weight gain, fat accumulation, inflammation, and insulin resistance hypoglycemic and anti-anemic action	
	phenolic compounds	hepatoprotective	[73]
	ganoderol B ganoderic acid, danderenic acid	antioxidant, anti-aging	[74]
		anti-tumor immunomodulation	
	polysaccharides and triterpenoids i.e., ganoderol B lucidumol D	anti-obesity	[30,75]
			[76,77]
		inhibit α-glucosidase	[78]
*Grifola frondosa*/Maitake, Hen of the woods	ether soluble/water soluble extract	decreases systolic blood pressure in SHR/ZFR animal models	[11,79,80,81,82,83,84]
	GF5000	decreases fasting serum glucose, total serum cholesterol and LDL-C level	
	SX-fraction	increases glucose uptake, decreases fasting serum glucose and insulin	
	polysaccharides	induces glucose consumption	
	Grifolan polysaccharide D-fraction/ MD-fraction of polysaccharides, galactomannan, heteroglycan	hypoglycemic, anti-inflammatory anti-tumor	[74,80,85]
	FXM (β-glucan)	anti-diabetic (decreases serum glucose and insulin level)	[86]
*Hericium erinaceus*/ Bearded tooth, Lion’s mane, yamabushitake	polysaccharides secondary metabolites	antimicrobial antioxidant	[11,87,88,89]
	enzymes (i.e., amylase, glucosidase), terpenoids chlorinated aromatic compounds, erinacerins	anti-hyperglycemic	[90,91]
		anti-hypercholesterolemic (LDL-C, TG, HMG-CoA, HDL-C) immunomodulatory	[92,93]
	benzopyrans	neuroprotective	
	benzofurans, ergosterol pyranones	anti-carcinogenic cytotoxicity	
*Inonotus obliquus*/Chaga	polysaccharides	anti-inflammatory	[94]
		increases sensitivity to glucose, reduces triglyceride levels, elevate the HDL-C/LDL-C ratio, restores the integrity of the glomerular capsules with decreased expression of TGF-β1	[95]
	triterpenoids, i.e., inotolactones A and B	inhibit α-glucosidase	[96]
*Lentinula edodes*/Shiitake	ergosterol, α-glucan, β-glucan	reduce triglyceride, and total cholesterol level increases plasma insulin and reduces blood glucose levels anti-inflammatory	[11,97,98,99,100]
	lentinan, eritadenina	anti-cancer	
*Lenzites betulina*/Wood-rooting fungi	α-glucan, β-glucan, β-glucan protein, galacturonic acid	antioxidant anti-hyperglycemic anti-inflammatory antiproliferative antibacterial	[94,101]
*Morachella esculenta*/ Morels	polysaccharides, proteins, vitamins and dietary fibers	improves composition of gut microbiota, improves gut barrier function reduces serum glucose, insulin, and pro-inflammatory cytokine levels	[102]
	polysaccharides (mannose, galactose, and glucose), phenolic compounds	antioxidant, anti-inflammation, immunoregulation, hypoglycemic, anti-hyperlipidemic	[103,104]
*Ophiocordyceps sinensis (Cordyceps sinensis)*/ winter worm, summer grass	cordycepin, adenosine, sterols, and many polysaccharides	anti-hyperglycemic, anti-inflammatory, antioxidant activities	[48]
*Panellus serotinus*/Mukitake	polysaccharides, phenolic compounds and antioxidants	Alleviates TG accumulation in the liver (increase lipolytic enzyme and suppress lipogenic enzyme activities) alleviate hyperinsulinemia enhance expression of adiponectin, improve insulin sensitivity suppress MCP1 and inflammatory cytokines, inhibit IKKβ	[105]
*Pleurotus djamor*	polysaccharides	antioxidant	[106,107]
*Pleurotus eryngii var. Ferulae*	polysaccharides, monosaccharides,	positive effect on gut microbiota (i.e., probiotics)	[41,108]
	mannogalactan heteropolysaccharides	decrease body weight, white adipose tissue weight, improved lipid profiles, glucose tolerance and insulin sensitivity	[30,109]
	SCFAs	increase GPR43 level in adipocytes	[110]
*Pleurotus ostreatus*/ Oyster mushroom	β-glucan	positive effect on gut microbiota (i.e., probiotics)	[41,108]
		improves postprandial glucose and lipid metabolism decreases MDA concentration and increases CAT, GSH-Px, and SOD activity	[111,112]
	terpenoids, heterocyclic amines, phenols, proteoglycan	anti-cholesterol, anti-cancer effect, anti-inflammatory, anti-diabetic	[113,114]
			[115]

**Table 2:** **Signaling pathways through which medical mushrooms improve insulin resistance**.

Targeted Process in IR	Involved Signaling Pathway	References
increased glucose uptake, inhibited increase in TG, insulin and weight gain	reverted insulin signaling through reactivation IRS-1/Akt pathway (induced GLUT4 translocation to plasma membrane)	[81,83]
increased glucose uptake	attenuated insulin resistance by inhibition of GSK-3 through activation of IRS-1/Akt pathway	[84]
anti-hyperglycemic effects	increased levels of p-AMPK and GLUT4 in muscle and adipose tissues	[113]
anti-hyperglycemic effects	up-regulated IRS-1, p-IRS-1, PI3K, Akt, pAkt, and GLUT4, and down-regulated p-JNK and p-p38 expression	[85]
anti-hyperlipidemic effects	upregulation of p-PI3K, p-Akt, GLUT4, and IRS-1 in the PI3K/Akt signaling pathway in the liver	[69]
inhibition of apoptosis in β-cells and inducing proliferation of β-cells	activation of Akt pathway through induction of IGF-2/IGF-1R expression by GLP-1	[141]
reduced endotoxin level, and inflammation	reverted insulin signaling through IRS-1 reactivation	[56,71]
decreased pro-inflammatory cytokine release from adipocytes	through suppressed TLR4/MyD88/NF-κB pathway	[80]
improve insulin resistance	induce insulin signaling by increased PI3K, IRS-1, and GLUT-4 levels	[58]
induce insulin sensitivity	SCFAs replacing carbohydrates decrease insulin/IGF-1 signaling	[106]
reduce insulin resistance	increased the mRNA expression of PI3K, p-Akt, Akt, p-38 MAPK, and p38 MAPK, and GLUT4 in rat skeletal muscle in vivo	[66]
increase glycogen content	activating GSK3 phosphorylation and GLUT4 translocation	[111]

## Supplementary Materials

The following supporting information can be downloaded at: https://www.mdpi.com/article/10.3390/ijms26020827/s1. References [12,14,23,25,97,204,205,206,207,208,209,210,211,212,213,214,215,216,217,218,219,220,221,222,223,224,225,226,227,228,229,230,231,232] are referring to Supplementary Materials.

## Abbreviations

ACC-1	acetyl-CoA carboxylase-1
ACS1	acyl-CoA synthetase-1
AMPK	AMP-activated protein kinase
C/EBP-α	CCAAT/enhancer binding protein-α
CAT	catalase
FABP4	fatty acid binding protein-4
FAS	fatty-acid synthase
FATP1	fatty acid transport protein-1
FFA	free fatty acid
FIP	fungal immunomodulatory proteins
GLP-1	glucagon-like peptide-1
GLUT4	glucose transporter type 4
GPR43	G-protein-coupled receptor 43
GSH	glutathione
GSH-Px	glutathione peroxidase
GSK-3	glycogen synthase kinase-3
HbA1c	hemoglobin A1c
HDL-C	high-density lipoprotein cholesterol
HMG-CoA	3-hydroxy-3-methylglutaryl-CoA
HOMA-IR	Homeostatic Model Assessment for Insulin Resistance
IgA	immunoglobulin A
IGF-1/IGF-2	insulin growth factor 1/insulin growth factor 2
IGF-1R	IGF-1 receptor
IKKβ	inhibitor of κB kinase β
IL-1β	interleukin-1 beta
IL-6	interleukin-6
IR	insulin resistance
IRS1/IRS2	insulin receptor substrate 1/insulin receptor substrate 2
IκB	inhibitor of κB
LDL-C	low-density lipoprotein cholesterol
LDLR	LDL receptor
MAPK	mitogen-activated protein kinase
MCP1	monocyte attracting protein 1
MCP1	monocyte chemoattractant protein 1
MDA	malonaldehyde
MetS	metabolic syndrome
MyD88	myeloid differentiation primary response 88
NF-κB	nuclear factor kappa B
NSPs	non-starch polysaccharides
PAI-1	plasminogen activator-inhibitor 1
PGC-1α	PPARγ co-activator 1α
PI3K	phosphoinositide 3-kinase
PPAR-γ	peroxisome proliferator-activated receptor-γ
PPAR-δ	peroxisome proliferator-activated receptor delta
PSA	prostate specific antigen
QoL	quality of life
RCTs	randomized control trials
RIP	ribosome inactivating proteins
ROS	reactive oxidative species
SCFAs	short-chain fatty acids
SHR	spontaneously hypertensive rats
SIRT1/SIRT6	sirtuin 1/sirtuin 1
SOD	superoxide-dismutase
SREBP-1c	sterol regulatory element-binding protein-1c
SXF	SX-fraction
T2DM	type 2 diabetes
TG	triglyceride
TGF-β1	transforming growth factor beta 1
TLR2/TLR4	toll-like receptor 2/toll-like receptor 4
TNF-α	tumor necrosis factor α
UVB	ultraviolet B irradiation
VDR	vitamin D receptor
ZFR	Zucker fatty rats
