# Supplementary material for: The Importance of Edible Medicinal Mushrooms and Their Potential Use as Therapeutic Agents Against Insulin Resistance
Source: Int J Mol Sci. 2025 Jan 19;26(2):827. doi: 10.3390/ijms26020827 (PMC11765957; doi:10.3390/ijms26020827)
Supplement: Supplementary file 1 [file ijms-26-00827-s001.zip › ijms-3384167-supplementary.pdf]

Supplementary Materials

Natural habitats and growing conditions of medical mushrooms

Mushrooms hold outstanding importance across multiple scientific fields. In this study, we examine their significance from the perspectives of nutrition science and health sciences, however in this section we detailed briefly some important information related to agriculture and food industry. In **Table 1** medical mushrooms with their natural habitat and growing conditions are listed.

Table S1. List of some medical mushrooms with their natural habitats and growing conditions

Name of the Medical mushroon (common name)	Natural habitat	Growing conditions	Ref.
Agaricus bisporus (White bottom mushroom)	Agaricaceae is grown in at least 70 countries worldwide	compost contains lignocellulosic material; mycelium grows in compost mass at 25 ºC for 16–18 days; pH to approximately 7.8	[1]
Agaricus brasiliensis (Agaricus blazei Murrill)	known from Brazil	higher temperature and need lightening compared to A. bisporus; secondary saprophyte – require fermented compost	[2]
Antrodia cinnamomea (Syn. *Antrodia camphorata*)	rare and valuable edible and medicinal fungus<br>Endemic to Taiwan - grow on rotting heartwood of Cinnamomum kanehirai (Hayata tree) at an altitude between 450 and 2000 m in a warm and humid climate and fertile soil	4 main artificial culture processes:<br>wood culture,<br>plate culture,<br>solid-state fermentation and<br>submerged fermentation	[3,4]
Flammulina velutipes (Enoki mushroom)	Found in temperate climate zones, the fungus follows a saprophytic lifestyle, growing on dead or dying woody plants such as elm, ash, beech, and oak. It is most commonly encountered in Europe, North America, and Asia	Hardwood logs, Sawdust, and rice bran. Fruits only at low temperature	[5,6]
Ganoderma lucidum (lingzhi or reishi)	Grows in subtropical and temperate climate regions such as Asia, Europe, Africa, and Americas. Root parasite of deciduous trees, its fruiting body appears at the base of trees or along the roots on the ground. Occasionally, it can also be found under pine trees.	hardwood logs, stumps, and sawdust<br>artificial cultivation takes a long time, and its quality is susceptible to environmental conditions, liquid- and solid-state fermentation are popular for the production of mycelia	[2,7,8]

	It is common in summer and autumn. It originated from the Dabie Mountains, which recorded in Compendium of Materia Medica.		
Grifola frondosa (Maitake)	Japan is especially hospitable for this mushroom, although it can also be found in the northern temperate forests of Asia, Europe, and eastern North America, as well. Roots of dead or dying deciduous hardwoods, such as Quercus, Ulmus, Acer, Nyssa, Larix and Fagus; sporadically also on Castanea, Prunus, Pseudotsuga and Pinus.	usually produced on substrate consisting of sawdust supplemented with wheat bran (various alternative solid state substrates have: beech sawdust, spruce sawdust) the optimized high-yielding formula included 73.125% corn cob, 1.875% rice straw, 23% wheat bran and 2% light calcium carbonate (CaCO3) (C/N = 48.40). (optimal growing conditions exist within a limited range of temperature, moisture, humidity)	[2,9,10]
Hericium erinaceum (Lion's mane, yamabushitake)	Rarely found in Europe and it has been placed on the red list in several European countries; in contrast, it is common in Japan and North America. Found on dead or dying deciduous trees belonging to genera Quercus sp., Fagus sp., Acer sp., Juglans sp., and Ulmus sp.	optimal temperature for mycelium growth was 25°C; best mycelium growth on substrates was observed at pH 6; most carbon sources, apart from lactose, promoted mycelium growth; alanine constituted the best source of nitrogen, histidine was the least advantageous	[11]
Inonotus obliquus (Chaga)	lives in certain parts of Europe and Asia at latitudes of 45–50 N as a parasite of birch trees	general-purpose mycology medium is potato dextrose broth; higher yield of the optimized formula of bioreactor culture: consist of malt extract, yeast extract, fructose, soluble starch, MgSO4, CaCl2.	[12,13]
Lentinula edodes (Shiitake)	Fagus longipetiolata forest in Shaanxi province, China is one of the natural location of LE. Outdoor wood log cultivation was initiated in 1939 and is still the dominant method in small family farms in mountainous regions in China.	Sterilized mixture of eucalyptus sawdust and olive mill cake wetted to 60% water content are packed into Unicorn Type M filter polypropylene bags. Bags then autoclaved at 121 °C for 1 h, and cooled to 25 °C for inoculation with the spawn. Culture then incubated at 25 °C for 21 days. For fruiting, the temperature is reduced to 16 °C with a relative humidity of 90%, daily illumination for 12 h by fluorescent 500 lx “Daylight” and air CO2 concentration of 600–800 ppm.	[2,14,15]
Ophiocordyceps sinensis (formerly Cordyceps sinensis) (winter worm, summer grass)	Endemic to the Tibetan Plateau, occurs in alpine meadow and shrub habitats from an altitude of 3,000 m up to the snow-line, optimum growth temperature at 18 °C. It is able to suffer	an expensive and rare species that is difficult to cultivate; in solid-state cultivation it is grown on grain-based substrates (germinated cereal grains, soybean seeds, and silkworm	[16-18]

	temperatures lower than −40°C in winter as a psychrophile, Lepidoptera: Hepialidae) and forms a worm-like sclerotium, from which the infected fungus forms one or more perithecial fruiting bodies or stromata.	pupae); can be cultured by submerged fermentation	
Panellus serotinus (mukitake)	It grows throughout the United States usually found growing on downed hardwood or conifer logs and overlapping, growing October through December.	mycelial growth was the fastest on Quercus acutissima and Q. mongolica sawdust media, the density was the highest on Quercus spp. sawdust-containing media;<br>conditions were 80~85 days of cultivation period after spawn inoculation, 10~11 days for primordial formation at 17~18°C, and 15~20 days for fruiting growth	[19,20]
Pleurotus eryngii var. Ferulae	Grow on the roots of Apiaceae plants such as Eryngium spp. and Ferula spp., and are generally distributed in Europe, Asia and Africa, especially being restricted to the Gobi Desert in Xinjiang, China.	non-composted range of substrate for *Pleurotus* are wheat straw, sawdust, paddy straw, soybean residue, groundnut skull, maize cob, bagasse, cornstalks, waste cotton, stalks and leaves of banana, rice bran, etc.	[21]
Pleurotus osreatus (oyster mushroom)	have been reported from the temperate areas of East and West Asia, North and South America, Europe, Africa and the tropical areas of Southeast Asia and growing on deciduous and coniferous trees, such as Fagaceae, Betulaceae, Salicaceae and Pinaceae.	traditional cultivation is on wood logs;<br>possible to produce on a wide variety of substrate: rice/wheat straw, waste cotton, sawdust, sugar beet pulp, or even banana leaves	[2,22]

Mushrooms can play a significant role in addressing global food supply challenges, and their importance in medicine has already been well-documented [23,24]. This is further evidenced by the substantial increase in mushroom cultivation across various continents. Between 1969 and 2009, global mushroom production increased nearly tenfold, according to the Food and Agriculture Organization of the United Nations (FAO). The largest growth occurred in China, the United States, the Netherlands, India, and Vietnam [25]. Additionally, mushrooms play a crucial role in soil remediation [6,26,27]. Environmental factors of nature affect both the size and quantity of mushrooms, which led humanity as early as the 600s to cultivate species such as *Auricularia auricula* (wood ear mushroom), *Flammulina*, and *Lentinula* [28,29]. Large-scale cultivation, however, only began in the early 20th century [29].

Recently, mushroom cultivation is gaining more importance for its sustainability, with a growing demand for more efficient, sustainable, and food-safe cultivation practices. Mushroom cultivation is one of the most cost-effective agricultural practices, as the substrate used for cultivation is primarily derived from various agricultural by-products such as wheat straw, rice straw, sawdust, and other plant residues rich in carbon and nitrogen, which often go to waste. This practice supports the natural recycling and optimal utilization of materials, contributing to a sustainable cycle [6].

Mushroom cultivation systems are designed to serve two main models, each targeting different goals. Low-investment models are tailored for local markets and direct consumption, while high-investment models focus on global markets and large-scale consumption [25]. For large-scale consumption the traditional log-based cultivation methods, which produced low yields, were replaced by substrates composed of specialized agricultural by-products, called compost, additionally, the cultivation period reduced from 150 days to just 40 days, leading to a 15- to 20-fold increase in productivity, as seen in Shiitake mushrooms [25]. Various compost preparations are available, which affect both the nutrient supply of mushroom cultivation and food safety [1,30]. Research has shown that the protein content of mushroom fruiting bodies is significantly affected by the chemical composition of the substrate and its carbon-to-nitrogen (C/N) ratio [31], as well as by the organic materials used such as by-products from agriculture, forestry, cotton production, and brewing industries [32].

2.1. Cultivation of medicinal mushrooms: comparison of traditional and solid-phase technologies.

Most medicinal mushrooms use traditional cultivation technology, where biomass is produced using long-term cultivation methods; i.e. *Ganoderma lucidum* (Reishi) fruiting bodies grow during 4 months, while *Grifola frondosa* (Maitake) during 6 months [33]. Traditionally, Shiitake and most other mushrooms were cultivated on various hardwood species. The process involved cutting natural logs in the fall, which were then inoculated with Shiitake spawn 15–30 days post-harvest. The spawn was supplied in the form of wooden plugs or sawdust. This method demonstrated very low productivity in terms of both wood and labour efficiency, and for a long time, no significant improvements were made to this technique [25].

However, rapid technologies bring benefits to both the pharmaceutical and dietary supplement markets. Solid-State Cultivation (SSC) offers a faster alternative i.e. *Ganoderma lucidum* mycelium biomass: 14 days and *Grifola frondosa* mycelium biomass: 18 days [33]. Rapid development in mushroom cultivation only began when several bio-innovations were

introduced in the late 1970s and early 1980s days [25]. One breakthrough in this cultivation method was the introduction of synthetic logs as a replacement for natural logs. Synthetic logs are made from sawdust, supplemented with millet and wheat bran, producing three to four times more mushrooms than natural logs in just one-tenth of the time days [25]. In environmentally controlled growing houses, factors such as temperature, humidity, light, and the moisture content of the logs can be regulated to achieve maximum yields. The main advantages of cultivating Shiitake on synthetic logs include consistent market supply through year-round production, higher yields, and reduced production cycle duration days [34].

References

1. Zięba, P.; Sękara, A.; Sułkowska-Ziaja, K.; Muszyńska, B. Culinary and medicinal mushrooms: Insight into growing technologies. *Acta Mycologica* **2020**, *55*.
2. Niksic, M.; Podgornik, B.B.; Berovic, M. Farming of Medicinal Mushrooms. *Adv Biochem Eng Biotechnol* **2023**, *184*, 29-76, doi:10.1007/10_2021_201.
3. Li, H.; Dai, J.; Wang, J.; Lu, C.; Luo, Z.; Zheng, X.; Lu, Z.; Yang, Z. Comparative Transcriptomic Analyses Propose the Molecular Regulatory Mechanisms Underlying 1,8-Cineole from Cinnamomum kanehirae Hay and Promote the Asexual Sporulation of Antrodia cinnamomea in Submerged Fermentation. *Molecules* **2023**, *28*, doi:10.3390/molecules28227511.
4. Wang, Y.; Zhang, Y.; Yuan, X.; Wang, J.; Yang, Y.; Zheng, Y. Transcriptome profiling of Antrodia cinnamomea fruiting bodies grown on Cinnamomum kanehirae and C. camphora wood substrates. **2023**.
5. Liang, Z.; Zheng, K.; Zhao, Q.; Shao, W.; Li, C.; Wang, J.; Ma, C.; Kang, W. Structural identification and coagulation effect of Flammulina velutipes polysaccharides. *Applied Sciences* **2021**, *11*, 1736.
6. Singh, C.; Pathak, P.; Chaudhary, N.; Rathi, A.; Dehariya, P.; Vyas, D. Mushrooms and mushroom composts in integrated farm management. *Res. J. Agric. Sci* **2020**, *11*, 1436-1443.
7. Szabó, L.G.; Babulka, P.; Fődi, A. A pecsétviaszgomba (*Ganoderma lucidum*). Available online: https://www.dxntermekek.info.hu/a-pecsetviaszgomba-ganoderma-lucidum/ (accessed on 10.11.2024).
8. Yang, Y.; Zhang, H.; Zuo, J.; Gong, X.; Yi, F.; Zhu, W.; Li, L. Advances in research on the active constituents and physiological effects of Ganoderma lucidum. *Biomedical Dermatology* **2019**, *3*, 1-17.
9. Gregori, A.; Švagelj, M.; Voglar, D.; Berovic, M. Growth characteristics and ergosterol content of Grifola frondosa in various solid-state substrates. *Chemical and Biochemical Engineering Quarterly* **2016**, *30*, 183-188.
10. Song, B.; Ye, J.; Sossah, F.L.; Li, C.; Li, D.; Meng, L.; Xu, S.; Fu, Y.; Li, Y. Assessing the effects of different agro-residue as substrates on growth cycle and yield of Grifola frondosa and statistical optimization of substrate components using simplex-lattice design. *AMB Express* **2018**, *8*, 46, doi:10.1186/s13568-018-0565-8.
11. Sokol, S.; Golak-Siwulska, I.; Sobieralski, K.; Siwulski, M.; Górka, K. Biology, cultivation, and medicinal functions of the mushroom Hericium erinaceum. *Acta Mycologica* **2015**, *50*.
12. Petre, A.; Ene, M.; Vamanu, E. Submerged cultivation of Inonotus obliquus mycelium using statistical design of experiments and mathematical modeling to increase biomass yield. *Applied Sciences* **2021**, *11*, 4104.

13. Lu, Y.; Jia, Y.; Xue, Z.; Li, N.; Liu, J.; Chen, H. Recent Developments in Inonotus obliquus (Chaga mushroom) Polysaccharides: Isolation, Structural Characteristics, Biological Activities and Application. *Polymers (Basel)* **2021**, *13*, doi:10.3390/polym13091441.
14. Drori, A.; Rotnemer-Golinkin, D.; Avni, S.; Drori, A.; Danay, O.; Levanon, D.; Tam, J.; Zolotarev, L.; Ilan, Y. Attenuating the rate of total body fat accumulation and alleviating liver damage by oral administration of vitamin D-enriched edible mushrooms in a diet-induced obesity murine model is mediated by an anti-inflammatory paradigm shift. *BMC Gastroenterol* **2017**, *17*, 130, doi:10.1186/s12876-017-0688-4.
15. Chiu, S.; Wang, Z.; Chiu, W.; Lin, F.; Moore, D. An integrated study of individualism in Lentinula edodes in nature and its implication for cultivation strategy. *Mycological Research* **1999**, *103*, 651-660.
16. Wang, W.; Wang, K.; Wang, X.; Yang, R.; Li, Y.; Yao, Y. Investigation on natural resources and species conservation of Ophiocordyceps sinensis, the famous medicinal fungus endemic to the Tibetan Plateau. *Protein Cell* **2018**, *9*, 671-673, doi:10.1007/s13238-017-0406-6.
17. Xia, E.H.; Yang, D.R.; Jiang, J.J.; Zhang, Q.J.; Liu, Y.; Liu, Y.L.; Zhang, Y.; Zhang, H.B.; Shi, C.; Tong, Y.; et al. The caterpillar fungus, Ophiocordyceps sinensis, genome provides insights into highland adaptation of fungal pathogenicity. *Sci Rep* **2017**, *7*, 1806, doi:10.1038/s41598-017-01869-z.
18. Chellapandi, P.; Saranya, S. Ophiocordyceps sinensis: A potential caterpillar fungus for the production of bioactive compounds. *Exploratory Research and Hypothesis in Medicine* **2024**, *9*, 236-249.
19. Woo, S.-I.; Ryoo, R.; Jang, Y.; Park, Y.; Jeong, Y.S.; Ka, K.-H. Mycelial culture and fruiting analysis of Panellus edulis strains collected in Korea. *The Korean Journal of Mycology* **2018**, *46*, 281-294.
20. Bonito Lab. Panellus serotinus. Available online: https://msu-prod.dotcmscloud.com/news/panellus-serotinus (accessed on 10.11.2024).
21. Dai, Y.; Sun, L.; Yin, X.; Gao, M.; Zhao, Y.; Jia, P.; Yuan, X.; Fu, Y.; Li, Y. Pleurotus eryngii genomes reveal evolution and adaptation to the Gobi desert environment. *Frontiers in microbiology* **2019**, *10*, 2024.
22. Li, J.; Han, L.-H.; Liu, X.-B.; Zhao, Z.-W.; Yang, Z.L. The saprotrophic Pleurotus ostreatus species complex: late Eocene origin in East Asia, multiple dispersal, and complex speciation. *IMA fungus* **2020**, *11*, 1-21.
23. Ahmad, R.; Riaz, M.; Khan, A.; Aljamea, A.; Algheryafi, M.; Sewaket, D.; Alqathama, A. Ganoderma lucidum (Reishi) an edible mushroom; a comprehensive and critical review of its nutritional, cosmeceutical, mycochemical, pharmacological, clinical, and toxicological properties. *Phytother Res* **2021**, *35*, 6030-6062, doi:10.1002/ptr.7215.
24. Thi Nhu Ngoc, L.; Oh, Y.K.; Lee, Y.J.; Lee, Y.C. Effects of Sparassis crispa in Medical Therapeutics: A Systematic Review and Meta-Analysis of Randomized Controlled Trials. *Int J Mol Sci* **2018**, *19*, doi:10.3390/ijms19051487.
25. Zhang, Y.; Geng, W.; Shen, Y.; Wang, Y.; Dai, Y.-C. Edible Mushroom Cultivation for Food Security and Rural Development in China: Bio-Innovation, Technological Dissemination and Marketing. *Sustainability* **2014**, *6*, 2961-2973, doi:10.3390/su6052961.
26. Rathod, M.G. Mushroom Farming: Exploring Varieties, Cultivation Strategies, and Endless Possibilities. **2023**.
27. Llanaj, X.; Toros, G.; Hajdu, P.; Abdalla, N.; El-Ramady, H.; Kiss, A.; Solberg, S.O.; Prokisch, J. Biotechnological Applications of Mushrooms under the Water-Energy-Food Nexus: Crucial Aspects and Prospects from Farm to Pharmacy. *Foods* **2023**, *12*, doi:10.3390/foods12142671.
28. Geösel, A.; Szabó, A. MODERN HORTICULTURE: MODERN MUSHROOM CULTIVATION TECHNOLOGIES. Available online: http://kerteszttananyag.hu/modern-mushroom-cultivation-technologies (accessed on 09.09.2024).
29. Gupta, S.; Summuna, B.; Gupta, M.; Annepu, S.K. Edible mushrooms: cultivation, bioactive molecules, and health benefits. *Bioactive molecules in food* **2018**, *1*, 1-33.

30. Singh, R.; Kim, J.; Shepherd Marion, W.; Luo, F.; Jiang, X. Determining Thermal Inactivation of Escherichia coli O157:H7 in Fresh Compost by Simulating Early Phases of the Composting Process. *Applied and Environmental Microbiology* **2011**, *77*, 4126-4135, doi:10.1128/AEM.02873-10.
31. Carrasco, J.; Zied, D.C.; Pardo, J.E.; Preston, G.M.; Pardo-Giménez, A. Supplementation in mushroom crops and its impact on yield and quality. *AMB Express* **2018**, *8*, 146, doi:10.1186/s13568-018-0678-0.
32. Jayaraman, S.; Yadav, B.; Dalal, R.C.; Naorem, A.; Sinha, N.K.; Srinivasa Rao, C.; Dang, Y.P.; Patra, A.K.; Datta, S.P.; Subba Rao, A. Mushroom farming: A review Focusing on soil health, nutritional security and environmental sustainability. *Farming System* **2024**, *2*, 100098, doi:https://doi.org/10.1016/j.farsys.2024.100098.
33. Berovic, M. Cultivation of Medicinal Mushroom Biomass by Solid-State Bioprocessing in Bioreactors. *Adv Biochem Eng Biotechnol* **2019**, *169*, 3-25, doi:10.1007/10_2019_89.
34. Sangeeta; Sharma, D.; Ramniwas, S.; Mugabi, R.; Uddin, J.; Nayik, G.A. Revolutionizing Mushroom processing: Innovative techniques and technologies. *Food Chem X* **2024**, *23*, 101774, doi:10.1016/j.fochx.2024.101774.
